# Exploring Self-Censorship and Self-Disclosure Among Clinical Medical Students with Minoritized Identities

**DOI:** 10.5334/pme.1661

**Published:** 2025-03-11

**Authors:** Vaishnavi Sankar, Tess M. Atkinson, Javeed Sukhera

**Affiliations:** 1 Baylor College of Medicine in Houston, Texas, US; 2 Institute of Living in Hartford, Connecticut, US; 3 Institute of Living, Hartford, Connecticut, US; 4 Institute of Living, Hartford Hospital in Hartford, Connecticut, US

## Abstract

**Introduction:**

Self-censorship and self-disclosure are two ways students negotiate and reconcile their personal identities with their burgeoning professional identities in order to succeed in the clinical learning environment. In this study, the authors explored how clinical medical students with minoritized identities navigate self-censorship and self-disclosure. Overall, the authors sought to better characterize perceived educational safety among minoritized medical students and identify strategies to better support trainees from diverse backgrounds.

**Methods:**

The authors utilized constructivist grounded theory methodology and conducted individual qualitative interviews from 2022–2024 with 16 clinical medical students in the United States who held one or more minoritized identities.

**Results:**

Participants viewed censorship as a mechanism for self-preservation in the context of biased and hierarchical learning environments, while disclosure served as a tool for connection and practicing authenticity. Navigating censorship and disclosure while holding the weight of minoritized identities proved challenging and affected learning. However, perceiving safety, trust, and invitation from others could facilitate this process. While participants noted the power of disclosure in improving patient care, many found it difficult to engage in disclosure while maintaining professional boundaries. Despite these challenges, participants found ways to use both censorship and disclosure to assert their identities and reclaim power in their identity narratives.

**Conclusions:**

Exploring self-censorship and self-disclosure provides valuable insight into perceived educational safety among students with minoritized identities. It is important for educators to be mindful of self-censorship and co-construct opportunities for disclosure with learners in order to promote inclusivity and equity within the clinical learning environment.

## Introduction

Safe and equitable learning environments are those in which all students feel comfortable taking risks and showing vulnerability without feeling shamed, targeted, or neglected [1,2,3]. Yet, sociocultural norms and hierarchical power structures within clinical learning environments may lead some students to aggressively self-monitor and maintain the image of a “perfect” medical student [1,2], [4,5]. This experience may be particularly salient for students with minoritized identities. Such students may disproportionately experience stereotype threat [4,6,7,8], which is the psychological phenomenon in which fear of confirming negative stereotypes about one’s background hinders academic performance [9]. Minoritized students may often feel pressured to combat microaggressions and hold themselves to higher standards than their peers to avoid negative stereotypes [4,7,8,11]. In addition, they may censor aspects of their identity due to fear of repercussions or differential treatment [4,7,8,11,12,13,14]. They may also compartmentalize their personal and professional identities in order to conform to dominant sociocultural norms [4,7,11]. Such hypervigilance to stereotype threat, pressure to conform, and identity compartmentalization adversely influences learning and erodes wellbeing [1,2,6,10].

Medical learners who are vulnerable to stereotype threat experience clinical learning environments differently from those who are less vulnerable. Such learners are unable to fully actualize their learning potential and authentically express aspects of their identity [11,12,15,16]. The term “identity capital” speaks to this baseline difference in comfort, suggesting that a student’s ability to assert and/or defend their identity is dependent on the relative assets of their various held identities [15]. Students with more “identity capital” typically hold identities which are well represented within their profession and are more concordant with their professional identities. Conversely, students with marginalized identities must negotiate and reconcile their held identities within established expectations of the medical profession [15,16,17]. Such students may undergo certain identity modifications, adapting their identity presentation to assimilate into the medical learning environment [4,8,15,18]. Meanwhile, some students may challenge the status quo by asserting their held identities as integral aspects of their professional identity. However, such expression often comes at a cost [19,20]. These identity negotiations occur in the context of interpersonal interactions and are shaped by the overarching dominant culture imposed by medical institutions [15,19,20,21].

Censorship and disclosure are two ways students negotiate and reconcile their personal identities with their professional identities. Self-censorship can be understood as any act of suppressing aspects about oneself. This can include censorship of speech, opinions, appearance, personality, or held identities. On the other hand, self-disclosure is the act of revealing information about oneself. Both self-censorship and self-disclosure can be conceptualized as tools to constrain or enable learning. For example, one study described physician self-disclosure as a powerful tool to promote educational safety, as it shows trust in the learner, softens hierarchy, and allows for openness in the learning environment [22]. Additionally, self-disclosure can be a mechanism through which individuals reframe their personal and professional identities on their own terms, reclaiming power and reducing self-stigma [23].

While these previous studies explored identity disclosure among healthcare professionals, few studies have specifically addressed how medical students experience and participate in self-disclosure. Furthermore, there is a gap in the literature highlighting experiences with censorship and disclosure among learners with multiple minoritized identities. In this study, we aimed to explore how medical students with one or more minoritized identities navigate censorship and disclosure in the clinical learning environment. We sought to understand how students approach censorship and disclosure within the context of their experienced social and intersectional identities as a reflection of perceived educational and psychological safety. We also aimed to better characterize how students use censorship and disclosure to construct their professional identities while navigating hierarchical norms within clinical learning environments.

## Methods

### Approach

We utilized constructivist grounded theory (CGT) to conduct a qualitative study exploring experiences with censorship and disclosure among clinical medical students who self-identified as holding minoritized identities. CGT is a useful approach which allows researchers to better understand a social process that is not well explained by established literature. CGT involves an iterative and theory-informed approach that builds upon existing literature while allowing for openness and flexibility. In this process, researchers engage in constant comparative analysis while continuing to collect and analyze qualitative data to inform a modern theoretical framework [24,25,26,27].

### Setting and Recruitment

Participants included clinical medical students who currently train at an accredited medical school in the United States or Canada. Consistent with our research question, we sought to sample theoretically for a diverse group of medical trainees with one or more minoritized identities who have participated in clinical rotations. Participants were recruited via social media using snowball sampling to include students from diverse geographic regions and academic institutions. Social media flyers were circulated on the social medial platforms Instagram and Twitter/X. Interested participants were directed to complete a short survey consisting of demographic questions regarding participant race, sexual orientation, gender identity, first-generation college student status, disability status, medical school affiliation, and year of medical training. Students who self-identified as belonging to one or more minoritized groups were invited to participate in a semi-structured interview scheduled on an individual basis with a member of the research team. Ethics approval was obtained from the supervising institution’s research ethics board.

### Data Collection, Analysis, and Reflexivity

Semi-structured qualitative individual interviews were conducted using a videoconference platform. Each interview began with answering questions and obtaining consent, followed by an introduction to key terms and exploratory questions to better understand each participant’s definitions, experiences, and perspectives of self-censorship and self-disclosure within the clinical learning environment (Appendix A). Initial versions of the discussion guide openly explored students’ experiences of navigating censorship and disclosure. Consistent with CGT, we iteratively adapted our interview guide as we engaged in constant comparative analysis.

Our team was composed of VS, first author and primary interviewer, who is a queer South Asian female medical student, TA who is a white female and master’s level research associate in social sciences, and JS who is a racially minoritized male psychiatrist and scientist in medical education. Our team addressed reflexivity through Walsh’s reflexivity typologies; personal, interpersonal, methodological, contextual, and collaborative. The identities and experiences of the interviewer may have guided the direction of each conversation and informed the interpretation of qualitative data. Dynamics between the interviewer and participant varied based on the nature of the individual interview. Some acknowledged the interviewer’s role as a medical student to contextualize their experiences. Others asked if any identifiable information would be shared with more senior members of the research team who worked within their medical institution. In these cases, participants were reassured that all information collected would be de-identified before analysis and would remain confidential. All participants were notified that they could withdraw at any time at no penalty to them. VS was involved in line-by-line coding of all transcripts and VS, JS, and TA participated in axial coding and discussion. Data was gathered until sufficient redundancy was achieved to build theory from our findings addressing the initial research question.

Overall, 16 individuals participated in our study. We invited participants to describe their identities in their own words. Participant demographics are listed below.

**Table:** 


**Gender Identity**	

Cisgender Women	11

Cisgender Men	3

Gender Non-Conforming	2

**Racial Identity**	

White	3

Black or African American	3

South Asian	7

Hispanic or Latino	2

Southeast Asian	2

**Disability Status**	

Mentally disabled	1

Physically disabled	1

Living with chronic illness	2

**Sexual Identity**	

Gay, Lesbian, Bisexual, or Queer	6

**Other Identifiers**	

Low-income	3

First-generation college student	1


## Results

Participants viewed censorship as a mechanism for self-preservation in the context of biased learning environments, while disclosure served as a tool for connection and practicing authenticity. Participants who felt the weight of holding their minoritized identity noted that the experience of balancing censorship and disclosure was challenging. However, perceiving trust, safety, and invitation from others could facilitate this process. Participants described how their navigation of censorship and disclosure affected their learning. Some struggled with a sense of detachment and withdrawal, while others noted the power of disclosure to improve patient care, foster professional development, and enhance psychological and educational safety.

### Behind the Mask: How Censorship and Disclosure are Experienced

Both censorship and disclosure served as mechanisms to negotiate and adapt aspects of one’s identity to fit the image of the ideal medical student. Participants described censorship as both a reaction to an established culture as well as a constant internal state of hyperawareness. Conversely, participants described disclosure as a way to assert identity and build authentic relationships. Students learned to balance censorship and disclosure as they navigated clinical clerkships, protecting their sense of identity safety while furthering their learning and growth.

Many participants viewed censorship as a *“self-conscious” P1* state of hypervigilance. The pressure to self-censor was stronger in settings where participants perceived a *“power dynamic” P10* and subsequently *“tailored themselves in a certain way” P4* to *“fit the space” P7*. However, censorship was also seen as a necessary survival mechanism driven *“by outside forces” P2*. As one participant stated, censorship is something that they are *“taught to do,”* to avoid making themselves *“a target” P3*. Most participants used censorship to control *“perceptions people have… on evaluations” P8* and protect themselves from their identities *“being used against [them]” P12*. Many students made the choice to uphold *“the status quo” P13* and *“be quiet, even if [they didn’t] necessarily agree”* with a situation, as anything they might say could *“put [them] back” P5*. While many participants pointed to *“constant evaluation” P8* as a driver of student censorship, one participant noted how censorship fundamentally depends on perceived in-group vs. out-group status.

*“There are two factors people really evaluate you on. One is how much they like you as a person. How funny are you? How nice are you? How much do they want to be around you? And the second one is how competent are you? How good is your patient care? And so if you can’t connect on the first one, then you have to do really well in the second one. Which you can rise to the occasion, but I do think that it adds an additional layer of stress.” P10*

In contrast to censorship, disclosure was experienced as a mechanism to foster socio-emotional connection. For many participants, disclosure enabled humanization, allowing students to *“bring closeness to a patient encounter” P5* and *“make [patients] feel safer” P12*. Additionally, disclosure was experienced as tool for connecting with residents, fellows, and clinical teachers and adding *“context” P15* to students’ motivations and aspirations. Ultimately, participants viewed disclosure as a way to practice authenticity and *“[show] your true self” P13*. However, several participants noted that disclosure was *“not something you can do all the time” P3* and requires a truly safe and comfortable learning environment. When they feel safe to do so, students used disclosure to be themselves without censorship, conformity, and hypervigilance. As one participant noted,

*“If I’m already deciding to self-disclose,… I feel comfortable enough in asking a lot more questions, because I’m not worried about what they’re thinking about me not only as a person but also as a learner.” P14*

### Walking the Tightrope: Navigating Between Censorship and Disclosure

Participants with minoritized identities delicately and cautiously navigated between censorship and disclosure to adjust and adapt to their clinical learning environments. Minoritized students described feeling less *“entitled” P8* to bring their full selves to their learning experiences compared to their peers. One participant described feeling as if they could only ever take up *“20% of the space”* they were in, as they were often *“the one listening”* and *“not really the one that’s talking” P9*. As one participant stated,

*“I want to take up more space with [my] identity, but I feel so hesitant to… it [leaves] me feeling an amplified sense of imposter syndrome and lack of self-confidence, and that translates in the clinical setting where I just … take less ownership of patients, or I don’t ask an attending a question… But I’ve seen my white counterparts not having any of that preamble in their thinking” P1*

Many participants attributed the pressure to self-censor to the perceptions and assumptions that come with their visible or embodied identity. Some participants described feeling pressure to undo stereotypes and responded by *“working twice as hard” P7* to show that they were *“just as capable if not more” P10* than their peers. However, some participants anticipated these assumptions and adapted their behavior to conform to others’ expectations in a more agentic way. One participant described how she *“played into”* the assumptions that she was *“timid”* and *“meek”* to *“adjust in ways that [made] her successful” P8*. Similarly, several racially minoritized participants noted that part of their motivation to self-censor came from anticipating and accommodating what they described as white discomfort. As one participant described,

*“You enter a space and you’re like, I may be the only Black person … maybe I need to act a certain way. Just so that I feel comfortable but also deep down it’s probably to make other people feel comfortable.” P7*

Several participants described how they censored themselves during clinical rotations to uphold perceived standards of professionalism. Black women participants censored by styling their natural hair to look *“less noticeable” P13*, while queer participants censored their gender presentation by choosing to *“not dress as colorful” P9*. Others described censorship as a form of ‘code switching’ with one Black woman participant describing how she sometimes speaks differently, changing her *“tone of voice”* or *“certain words,”* to not portray *“specific stereotypes people might have against Black females” P13*. In this way, participants used censorship to diminish parts of their identity which they perceived as not belonging in professional clinical spaces. As a queer identifying Latino participant stated,

*“I’ll change the way I present to a person… just trying to speak in a more masculine voice, in a deeper voice, I’ll tend to do that. Something I’ll try to do I think is avoid speaking Spanish unless I need to…I’m thinking about trying to say everything, right, and in English, and I’m speaking very slowly to make sure I don’t mess up. Part of that is so that I come across as I guess, professional or put together.” P14*

Despite the pressure to self-censor, participants were able to engage in disclosure when they perceived that it was safe to do so. Some participants stated they typically started each rotation with the *“baseline” P11* of censorship, disclosing in *“increments to show more parts” P9* of themselves based on how their educators *“show that they can share” P11*. Some described using small disclosures to *“test the waters” P2 or “build in checks” P16*. Many participants described identity disclosures as tiered, and navigated clerkships by disclosing identities they perceived as holding more *“privilege in the healthcare system” P5*. As one participant stated,

*“Outwardly you see me as a cisgender South Asian woman and I definitely feel like overall I am very privileged … because that’s also the most visible part of my identity. The more minoritized identities are the parts of my identity that I hide and don’t disclose.” P5*

However, balancing these various identities led to some participants describing a sense of dissonance between their held and performed identities. One participant described this dissonance as having *“two wolves in [their] head.”* As a queer immigrant who grew up in a low-income household, they described one part of themselves wanting to visibly show pride in their queer identity, while the other part struggled with the fear of *“screw[ing] up [their] chance” P12* or losing access to privilege.

Disclosure amongst medical learners was often selective and strategic. Some participants described disclosing aspects of their identity which are perceived as *“productive”* or *“professional” P1*. As one participant stated,

*“I tend to share very neutral things about myself. Neutral hobbies, neutral tidbits about growing up and not so much the lived experiences of growing up—how being a Black woman has affected me. I think always for me, you have to keep in mind the extra questions they could ask.” P3*

Participants used selective disclosure to avoid entering into *“uncomfortable” P3* conversations that they *“don’t have the bandwidth” P6* to engage in. For example, one participant noted how the *“invasive questions”* people would ask about her disability after disclosing accommodations made her want to *“push through” P16* and suffer in silence.

Censorship and disclosure were largely influenced by perceived power and hierarchy within clinical learning environments. In teams with rigid and hierarchical structures, *“respect is shown one way” P15*, creating an environment in which students don’t *“volunteer” P6* information about their personal identities. Being on the *“bottom” P4* made it “*hard to initiate those conversations” P15*. Conversely, on teams in which students were given power and had more autonomy, they felt they had *“space to disclose things about [themselves]” P9*. Witnessed disclosures from residents or faculty also affected student disclosure. Some participants noted that there are *“elements of humanization” P1* that occur when attendings engage in self-disclosure that make them appear more *“trustworthy”* and willing to *“be vulnerable” P3*. In this way, some students viewed disclosure as earned, as there are *“certain parts”* of their identity that others are not *“entitled to know” P5*. While students interpret these various signals to determine if an environment is safe for self-disclosure, one participant noted that there is an *“early bifurcation” P8* in whether or not she perceives a team to be safe for disclosure.

*“I think right off the bat, the tone is set in the different dynamics of how inclusive or exclusive I perceive the team to be. When we’re doing our introductions, and people give a little bit of background about their identities… That kind of sets the tone for how much I’m willing to disclose on my end. And ultimately, how much more comfortable I feel on that team.” P8*

Thus, self-disclosure was experienced as both enabling and resulting from educational safety, allowing learners to surpass hierarchical roles and assert belonging within the clinical learning environment.

### On the sidelines: Censorship, Disclosure, and Implications for Learning

Participants described censorship and disclosure as intrinsically related to their learning. Censorship was associated with factors that could adversely influence learning such as detachment, distraction, and psychological stress, while disclosure was perceived as a mechanism to enhance learning through connection and community. Participants described how the pressure to censor pushed them to the *“sidelines” P8*. One participant mentioned how she was often perceived as *“not engaged”* even though she felt *“interested enough”, “asked questions”*, and *“did the tasks [she] was supposed to do” P3*. One participant described how the hypervigilance to self-censor affected her participation in the learning environment.

*“There’s always that baseline level of, what words are going to be used to describe me down the line? And what words do I want to describe me down the line? And that just kept playing into the cycle for me to stay as invisible as possible. As long as I do the work that’s expected of me, that’s how I can be memorable.” P8*

Many participants described censorship as associated with *“social anxiety” P9* and perfectionism. For example, Black participants recognized that they might need to work harder to be placed at the *“same level as someone who isn’t Black” P7*. Another noted, *“when I talk, it has to be the most articulate” P9*. This pressure to perform perfection takes a mental toll and *“puts [minoritized students] behind” P3*. One participant noted that the time she spent wondering about others’ assumptions is time that could’ve been spent *“learning … about other things” P7*. Another similarly remarked that *“the more marginalized identities you hold, the more extra space you’re keeping in your brain to react and decompress from the experiences that you’re having” P3*.

In contrast to censorship, disclosure was associated with enhancing safety and fostering connection. Several participants described how sharing experiences with residents and attendings allowed them to *“build bonds” P6* and *“contribute to the team dynamic” P1* in a way that surpassed hierarchical relationships. One participant noted how if she developed rapport with residents early on, she felt more comfortable pursuing learning opportunities rather than *“giving off the illusion of learning” P8*. This *“baseline comfort” P1* established through disclosure can be used to enhance educational safety and ultimately improve patient care. Several participants remarked that engaging in self-disclosure during patient encounters allows patients who *“distrust” P11* medical providers to *“feel more comfortable opening up” P8*.

However, some participants found it difficult to engage in meaningful self-disclosure with patients, as they felt *“expected to come to patient encounters as… identity-less” P5*. While some participants viewed this aspect of clinical medicine as a *“good form of censorship” P2*, others expressed that the heavy emphasis in medical school on *“being professional”* and *“maintaining boundaries”* ran counter to their innate desire to be *“human”* and *“vulnerable” P1* with patients. Several participants described the dehumanization they experienced throughout medical training as resulting from educational systems which don’t allow for self-disclosure and *“shut off empathy” P8*. Some students struggled to detach themselves from patients’ experiences while holding their lived experiences, as they know what it’s like *“on the other side” P5*. As one participant described,

*“I think there’s an expectation of a certain amount of detachment…It almost feels like if one were to talk about [their identities] they would no longer be detached or impartial or the kind of things that we expect from healthcare providers.” P5*

Despite these challenges, some participants found ways to use disclosure to assert their identities and reclaim power in their identity narratives. As one participant stated, “*declaring your identity and framing it on your own terms is sort of empowering. Because you have the choice to shape how that’s going to be perceived.” P2*. Students declare identity in various ways, namely through advocacy and community building. One participant described how advocacy acted as a form of public disclosure as it made them *“the most visibly disabled person in [their] medical school” P16*. Some participants attempted to build community by actively seeking mentors who they *“[could] relate to” P1*. However, participants noted that attendings were *“not very forthcoming” P1* about their identities. Additionally, while building community helped minoritized students feel like they *“belong in this space” P15*, some participants described how peer sharing could perpetuate the *“expectation”* to experience *“microaggressions” P7* and reinforce censorship. One participant described the impact of hearing her friends’ experiences on clinical rotations.

*“It kind of negatively reinforces me to [not] try to even stand up for myself or to say certain things. Because I worry that it’ll be met with the same negative consequences that friends with similar identities have had. And even though we need that debriefing, maybe it would have been better for myself to just not hear it.” P8*

Overall, we found that exploring self-censorship and self-disclosure among medical students with minoritized identities provides valuable insights into the perceived safety of clinical learning environments. Educational settings in which students with minoritized identities feel a need to self-censor are perceived as hindering optimal learning, as students may experience undue pressure to excel, increased cognitive load, and subsequently withdraw from the learning environment. While navigating this difficult terrain, students find ways to use censorship and disclosure to form their professional identities and build relationships with patients and peers. Students also use advocacy and community building as forms of public disclosure, empowering themselves in their identities and building their sense of identity safety.

## Discussion

In this study, we investigated experiences of censorship and disclosure to better characterize the current climate of clinical medical education for students with minoritized identities and identify strategies to promote inclusivity and educational safety within the clinical learning environment. We found that students viewed censorship as adversely impacting their learning and disclosure as a potential tool to enhance learning and patient care. Our findings are largely consistent with existing literature on identity threat [28,29], suggesting that students who hold one or more minoritized identities disproportionately experienced the adverse effects of self-censorship, including hyperawareness to stereotype threat, disengagement from the learning environment, increased mental burden, and depersonalization [8]. However, our findings also extend existing knowledge by providing insights into the unique ways that minoritized learners navigate censorship and disclosure to enhance opportunities for growth and learning.

Participants used censorship and selective disclosure as strategic tools to reframe their identities and advance their professional goals. Some students found power in decentering their personal identities as they formed professional identities, as they worried that self-disclosure could be stigmatizing [30]. Such identity negotiations occurred within the context of experiences with bias, differential treatment, and microaggressions. Traditional theories of professional identity formation characterize the dissonance that many minoritized individuals may experience as resulting from a system which was designed by and for the dominant group [31]. Our data adds nuance to this idea, suggesting that minoritized students respond to dissonance by developing new ways of conceptualizing their multiple identities to adapt and thrive in their environments. Students may opt to maintain strict separations between their personal and professional selves and leverage censorship to avoid stereotypes about their held identities. Certainly, medical institutions must address the root causes of a pervasive culture that normalizes and anticipates bias. However, it is also important to acknowledge that students with minoritized identities may not want to self-disclose and find solace in censorship.

While self-disclosure can pose many benefits for medical learners, it is fraught with tensions and requires considerable emotional and cognitive effort. Our study contributes to the growing body of evidence that self-disclosure can enhance the therapeutic alliance, especially among patients who may distrust the healthcare system. Studies have shown that physicians utilize various forms of disclosure to build patient rapport, and physician self-disclosure can increase perceived empathy of physicians among patients [32,33]. However, we also learned that some medical learners struggled to engage in self-disclosure during clinical encounters and many engaged in censorship to maintain appropriate patient boundaries and uphold perceived standards of professionalism. Some participants perceived identity disclosures as not belonging in the clinical setting, and found it difficult to censor their identities while forming meaningful connections with patients. As medical students and residents experience empathy decline throughout their training, self-disclosure may be a tool to help humanize patient interactions and find meaning as healthcare providers [34].

Nevertheless, many participants felt a tension between emulating professionalism and practicing authenticity, consistent with existing literature exploring self-disclosure among resident physicians [35]. Participants described the dehumanization they experienced as a symptom of the medical training system. However, they viewed disclosure as a way to acknowledge personhood within a society that expects healthcare providers to serve selflessly, expanding upon existing data that medical students experience identity safety when they perceive others to recognize their individuality [6]. While these experiences may be common among all medical students regardless of identity, our findings suggest that self-disclosure can play a role in breaking the cycle of depersonalization and detachment characteristic of burnout, which minoritized medical students are at a greater risk of experiencing [36].

As we aim to build safe and inclusive learning environments, educators must be mindful of student self-censorship and co-construct opportunities for disclosure with learners. Consistent with existing literature, when educators model vulnerability by engaging in self-disclosure, learners feel empowered to identify their shortcomings and pursue learning opportunities [35]. Similarly, inviting reciprocal disclosure builds trust between learners and educators and contributes to learning environments that normalize vulnerability and embrace humanism [35,37,38,39,40]. Our data suggests that for students with minoritized identities, making space for self-disclosure in the clinical learning environment may help students combat responses to stereotype threat and integrate their personal and professional identities. In addition, we found that clinical teams in which medical students were given autonomy promoted student self-disclosure, which expands upon existing literature that perceiving agency promotes identity safety among medical students and allows for the development of professional identity [6,41]. Professional relationships built through disclosure allow for shared vulnerability and improved learning experiences [35]. Such moments can be made visible by increasing attention to the power of disclosure while creating space for learners to navigate potential boundaries in a thoughtful and measured way.

Mentorship, community building, and advocacy serve as powerful avenues which allow for student self-disclosure. However, multiple students noted that physicians rarely spoke openly about their identities. Existing literature suggests that racially minoritized physicians may not feel as able to bring their racial identities to their professional roles compared to other racially minoritized healthcare professionals [42]. Further investigation into perceptions of self-disclosure among minority physicians may provide greater insight into how medical institutions can support clinical faculty in engaging in disclosure. Community building and advocacy appeared to be protective factors which helped students with minoritized identities find belonging. However, several participants expressed how peer sharing could reinforce beliefs about the clinical learning environment that promoted censorship. This is consistent with the *peer socialization hypothesis*, which proposes that in-group peer sharing may reciprocally enhance perceptions of discrimination by centering a perceived shared reality [43]. While providing structured safe spaces for minoritized students to engage in disclosure is incredibly important, institutional interventions may benefit from involving peer facilitators who can redirect conversations which perpetuate harmful narratives [44,45,46].

Despite these recommendations, this study has limitations. While we aimed to center the experiences of clinical medical students with multiple marginalized identities, we were not able to represent certain identities in our participant pool. Given that we relied on social media recruitment and snowball sampling, participants came from medical institutions that mirrored the geographic locations and affiliations of the research team. In addition, while our study highlighted the experiences of students who self-identified as minoritized, the focus of our work was not on the experiences of students who did not identify as such. Future research could aim to explore experiences of censorship and disclosure among diverse groups of students who experience or perceive minoritization regardless of how they identify their demographic categories or social identities. For example, stratifying samples using stereotype vulnerability or experiences of discrimination may shed further light on how minoritization relates to censorship and disclosure. In addition, further examination of the factors which enable disclosure among clinical faculty and resident physicians with minoritized identities may also help us identify areas for improvement.

Overall, our study highlights the experiences of medical students with minoritized identities and investigates the complex dynamics which affect how they navigate clinical clerkships. This study also better characterizes how students with multiple marginalized identities negotiate their intersecting identities to adapt to their learning environments. We establish the role of self-censorship and self-disclosure among minoritized medical students in their socialization to the medical workforce and development of professional identity. We believe that our study provides important insights into factors which promote educational safety for students with marginalized identities. In addition, we identify opportunities for intervention which medical institutions can consider as they grow to support medical trainees from diverse backgrounds.

## Additional File

The additional file for this article can be found as follows:

10.5334/pme.1661.s1 Appendix A. Interview Guide.
