# Supplementary material for: Exploring Self-Censorship and Self-Disclosure Among Clinical Medical Students with Minoritized Identities
Source: Perspect Med Educ. 2025 Mar 11;14(1):107–17. doi: 10.5334/pme.1661 (PMC11908417; doi:10.5334/pme.1661)
Supplement: Appendix A. — Interview Guide. [file pme-14-1-1661-s1.pdf]

Interview Guide
Answer these questions based on your experiences in clinical rotations

1) What does self-censorship mean to you?
   a) Can you think of certain situations in which you chose to engage in self-censorship? Can you tell me more about that?

2) What does self-disclosure mean to you?
   a) Can you think of certain situations in which you chose to engage in self disclosure? Can you tell me more about that?

3) In your clinical experiences, have you ever had a supervisor or physician mentor with a similar identity to yours?
   a) Can you describe what that was like for you?
   b) Can you describe your behavior around this person?

4) Is there anything else you would like to share that I haven't already asked about?
